# Carboxylesterase 1 Gene Duplication and mRNA Expression in Adipose Tissue Are Linked to Obesity and Metabolic Function

**DOI:** 10.1371/journal.pone.0056861

**Published:** 2013-02-28

**Authors:** Martin Friedrichsen, Pernille Poulsen, Jørgen Wojtaszewski, Peter Riis Hansen, Allan Vaag, Henrik Berg Rasmussen

**Affiliations:** 1 Department of Diabetes and Metabolism, Rigshospitalet, Copenhagen, Denmark; 2 Department of Nutrition, Exercise and Sports, University of Copenhagen, Copenhagen, Denmark; 3 Global Development, Novo Nordisk A/S, Bagsværd, Denmark; 4 Department of Cardiology, Copenhagen University Hospital Gentofte, Hellerup, Denmark; 5 Research Institute of Biological Psychiatry, Mental Health Centre Sct. Hans, Copenhagen University Hospital, Roskilde, Denmark; Consiglio Nazionale delle Ricerche, Italy

## Abstract

**Context and Aims:**

Carboxylesterase 1 (CES1) appears to play an important role in the control of the metabolism of triglycerides and cholesterol in adipocytes and other cell types including hepatocytes. Therefore, it is relevant to gain insights into the genetic versus non-genetic mechanisms involved in the control of *CES1* mRNA expression. Here, we investigated *CES1* mRNA expression level in adipose tissue and its association with measures of adiposity and metabolic function in a population of elderly twins. Furthermore, the heritability of *CES1* mRNA expression level in adipose tissue and the effect of *CES1* gene duplication were assessed.

**Methodology:**

A total of 295 monozygotic and dizygotic twin subjects (62–83 years) with (*n* = 48) or without (*n* = 247) type 2 diabetes mellitus were enrolled in the study. They were subjected to a standard oral glucose tolerance test and excision of abdominal subcutaneous fat biopsies during the fasting state. Levels of *CES1* mRNA and copy number of the gene were assessed by quantitative PCR.

**Results:**

*CES1* mRNA expression level in adipose tissue was positively associated with body-mass index (*P*<0.001), homeostasis model assessment-insulin resistance (*P* = 0.003) and level of fasting glucose (*P* = 0.002), insulin (*P* = 0.006), and triglycerides (*P* = 0.003). The heritability for the expression of *CES1* mRNA in adipose tissue was high. *CES1* gene duplication was positively associated with insulin sensitivity (*P* = 0.05) as well as glucose tolerance (*P* = 0.03) and negatively associated with homeostasis model assessment-insulin resistance (*P* = 0.02). Duplication of *CES1* was not linked to mRNA level of this gene (*P* = 0.63).

**Conclusion:**

CES1 mRNA in adipose tissue appears to be under strong genetic control and was associated with measures of metabolic function raising the possibility of a potential role of this enzyme in the development of type 2 diabetes mellitus. Further studies are needed to understand the potential effect of *CES1* gene duplication on adipocyte and whole-body metabolic functions.

## Introduction

Adipose tissue is the major site for storage of cholesterol and triglycerides in the body [1], [2] The hydrolysis and esterification of lipids in this tissue are closely regulated to maintain a tight balance between lipid release, storage, and trafficking [3]. Disturbance of this balance may lead to insulin resistance, increased plasma levels of triglycerides, and obesity, i.e. components of the metabolic syndrome [3].

Several enzymes are implicated in the lipid metabolism in human adipose tissue [4]. This includes carboxylesterase 1 (CES1) that seems to play an important role in the hydrolysis of cholesteryl esters and triglycerides [5]. This enzyme uses a hydrolysis mechanism with release of the alcohol substituent from the substrate and formation of a fatty acyl-enzyme intermediate as the first step followed by a second step consisting of reaction of the intermediate with water and release of the acyl group-containing molecule [6]. Apparently, CES1 also possesses cholesterol transferase activity that enables formation of cholesteryl esters in the abundance of free cholesterol [7]. Hence, the enzyme appears to be involved in the de-esterification as well as transesterification of lipids.

A role of carboxylesterases in the lipid metabolism in humans is supported by observations from animal models [8]–[10]. One such study showed that the hydrolysis of triglycerides in adipose tissue was severely decreased in mice with global inactivation of Ces3, recently annotated as Ces1d1, which is the murine ortholog of human CES1 [8]. Moreover, liver specific inactivation of Ces1d1 in mice has been shown to be associated with significantly decreased levels of very low density lipoprotein-triglycerides and -cholesterol in plasma and moderately increased liver triglyceride levels suggesting an important role of hepatic Ces1d1 in the lipid metabolism [9].

Because CES1 has a potentially significant role in the lipid homeostasis, and its activity has been suggested to be primarily regulated on the transcriptional level [11], the amount of *CES1* mRNA in adipose tissue may serve as an intermediate phenotype providing important information about the mechanisms leading to type 2 diabetes mellitus (T2DM). So far, the expression of *CES1* mRNA in T2DM has not been examined. However, it has been suggested that the expression of *CES1* mRNA in human adipose tissue is correlated with measures of adiposity and metabolic function including waist circumference, homeostasis model assessment-insulin resistance (HOMA-IR), triglyceride level, and plasma insulin level [12]–[14]. In contrast, findings of a relationship between *CES1* mRNA level and other measures of metabolic function such as body-mass index (BMI) and total cholesterol level are inconsistent, possibly reflecting limited statistical power [13], [14] and inclusion of study participants with malignant diseases [14].

Since CES1 may play an important role in the mechanisms leading to dysregulation of lipid and glucose metabolism and eventually to T2DM [13], [14], the heritability of the expression level of *CES1* mRNA in adipose tissue is of scientific interest. Yet, this heritability has not been determined. Therefore, the relative contribution of genetic and non-genetic variation to the expression of *CES1* mRNA in subcutaneous adipose tissue remains unknown.

A variety of genetic variations may influence *CES1* mRNA expression. One such variation is duplication of the gene [15] that may result in increased production of *CES1* mRNA and thus increased CES1 activity albeit the “daughter” copy of the gene has been reported to be transcribed at a lower level than the “mother” copy [16]. Since pseudogenes can affect the mRNA levels of their protein-coding counterparts [17], another inherited factor that could potentially modulate the level of mRNA of *CES1* is carboxylesterase 1 pseudogene 1 (*CES1P1*), located in the vicinity of *CES1* [18].

In the present study we examined a unique study population of phenotypically well-characterized elderly monozygotic and dizygotic twin subjects (*n* = 295 individuals) with the aim of providing new information about basal mechanisms underlying T2DM by: 1) assessment of the relationship of *CES1* mRNA expression level in subcutaneous adipose tissue with age, gender, measures of glucose and lipid metabolism, and *CES1P1* mRNA expression level; 2) determination of the heritability of *CES1* mRNA expression in adipose tissue; 3) assessment of the relationship of *CES1* copy number with *CES1* mRNA expression in adipose tissue and measures of metabolic function in healthy and T2DM subjects.

## Materials and Methods

A total of 295 monozygotic (MZ) (*n* = 125; 48 complete pairs and 29 single twins) and same-sex dizygotic (DZ) (*n* = 170; 55 complete pairs and 60 single twins) elderly Danish twin subjects (62–83 years) were recruited as previously described [19], [20]. Glucose tolerance status was determined employing a standard 75-g oral glucose tolerance test (OGTT). The glucose tolerance status was defined according to the WHO 1999 criteria [21] and ranged from normal glucose tolerance (NGT, *n* = 169) over impaired glucose tolerance (IGT, *n* = 78) to overt T2DM (*n* = 48). Twenty-two subjects had known T2DM and were treated with diet or glucose-lowering medication. An informed consent form was signed by all subjects prior to their inclusion in the study. The signed forms were kept by the principal investigator for documentation purposes. The study with its consent procedure was approved by the Committees on Biomedical Research Ethics for the Capitol Region in Denmark and conducted in adherence with the guidelines of the Helsinki Declaration.

### Clinical Examination

Weight and height as well as waist and hip circumference were measured, and BMI was calculated. Fasting blood samples were analyzed for serum triglyceride and total cholesterol levels using commercial kits from Boerhinger Mannheim (Mannheim, Germany). Insulin resistance was assessed using HOMA-IR: (fasting plasma insulin*fasting plasma glucose/22.5)*0.144 [22]. The composite whole-body insulin sensitivity index (ISI)_composite_ was determined as follows: 10,000/sqrt([fasting glucose*fasting insulin]*[mean glucose_OGTT_*mean insulin_OGTT_]) [23].

*Tissue biopsies.* Subcutaneous adipose tissue biopsies were taken from the abdomen in a subgroup of the study population (*n* = 226). This was done under local anesthesia (lidocaine) using a Bergström needle with suction applied. Specimens were quickly blotted onto filter paper and frozen in liquid nitrogen.

### Gene Expression

Total RNA was extracted using TRI reagent (Sigma-Aldrich, St. Louis, MO) as described previously [24]. cDNA was synthesized using QuantiTect Reverse Transcription Kit according to the manufacturer’s recommendations (Qiagen, Valencia, CA). We used the standard curve method for quantification of mRNA by TaqMan®-based PCR on an ABI Prism 7900 HT system from Applied Biosystems, now Life Technologies Corporation (Carlsbad, CA). Using reagents also purchased from this company, we determined the mRNA levels of *CES1* in 206 individuals (assay identification Hs00275607_m1), while that of *CES1P1* only were determined in 159 individuals due to technical issues (assay identification Hs00750233_s1). The mRNA levels were normalized to that of *PPIA*, cyclophilin A, (part number 4326316E from Life Technologies Corporation) and expressed in arbitrary units. Average Ct values were ∼25 for *CES1* and *PPIA* and ∼33 for *CES1P1*. The lower expression of *CES1P1* and thus larger variation between the duplicates allowed for fewer duplicates to pass the criteria of a CV% <1%.

### Copy Number Determination of CES1

DNA was purified from leukocytes employing Autopure LS® according to the manufacturer’s recommendations (Qiagen GmbH, Hilden, Germany). The copy number determination was performed using real-time PCR followed by comparison of the amounts of gene-specific amplicon to that of a reference gene. For this purpose commercially available fluorescent-labeled reagents were acquired and applied according to the guidelines of the manufacturer (Life Technologies Corporation). These reagents targeted a region in exon 11 of *CES1* (assay identification Hs00139541_cn). The gene encoding the ribonuclease P RNA component served as reference gene. Calculation of the gene copy number was accomplished using CopyCaller v 1.0, a program developed by the manufacturer of the assay reagents. In the event of disagreement between duplicate samples, the analysis was repeated. The observed genotype proportions were compared with those expected under Hardy-Weinberg equilibrium using chi-squared test.

### Statistical Methods

All statistical tests were performed using SAS (version 9.2, SAS Institute, Cary, NC). Data were presented as mean±SD. *P<*0.05 was considered significant. Spearman’s rho was used to evaluate the correlation between continuous variables. Multiple regression analyses allowed for assessment of the effect of *CES1* copy number and *CES1* mRNA level, while adjusting for twin and zygosity status as well as for additional contributing variables, including age, sex, and BMI [25]. All response variables were log-transformed to avoid skewness of the residuals. This resulted in effects that expressed percentage-wise and not absolute changes in the response variable. Since *CES1* mRNA level was expressed in arbitrary units, the association of this variable with others was calculated per doubling from its average. Doubling of the *CES1* mRNA level was within 2 SD, which was considered to be biologically plausible. The heritability coefficient, often abbreviated as h^2^, expresses the proportion of the total variation of a trait attributable to genetic variation. In a twin population the heritability coefficient can be calculated as the degree to which MZ twins are more similar than DZ twins [26]. It was determined as twice the difference of the intra-class correlation coefficients of MZ and DZ twins (h^2^ = 2[r_MZ_−r_DZ_]). A heritability coefficient approaching 0 indicates very limited genetic influence, whereas a heritability coefficient close to 1 indicates that the trait is under strong genetic control.

## Results

### Clinical Characteristics

The clinical characteristics of this population have been described in detail previously [20].

**Figure 1:**
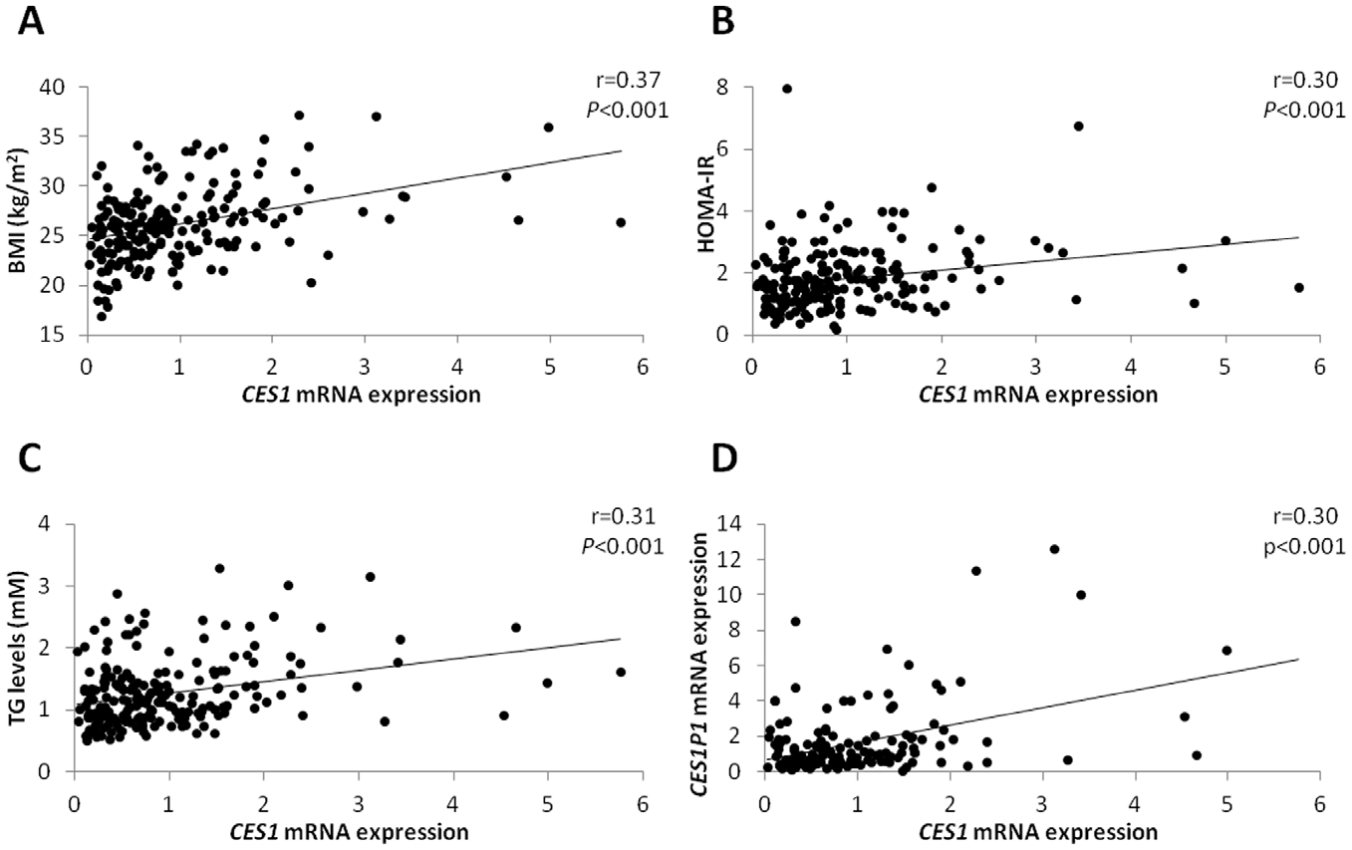
Correlation between *CES1* mRNA expression level and selected variables. Correlation of *CES1* mRNA level with body-mass index (BMI) (A, *n* = 204 twin subjects), homeostasis model assessment-insulin resistance (HOMA-IR) (B, *n* = 203 twin subjects), fasting plasma triglyceride levels (C, *n* = 205 twin subjects), and *CES1P1* mRNA level (D, *n* = 147 twin subjects), respectively, are shown. Due to technical reasons the level of *CES1P1* mRNA could not be determined in a relatively large subpopulation of the twin subjects (for explanation, see ”Materials and Methods“). *CES1*: Carboxylesterase 1 gene; *CES1P1*: carboxylesterase 1 pseudogene 1; TG: triglyceride. Spearman’s rho correlation coefficients and the corresponding *P*-values have been included.

**Figure 2:**
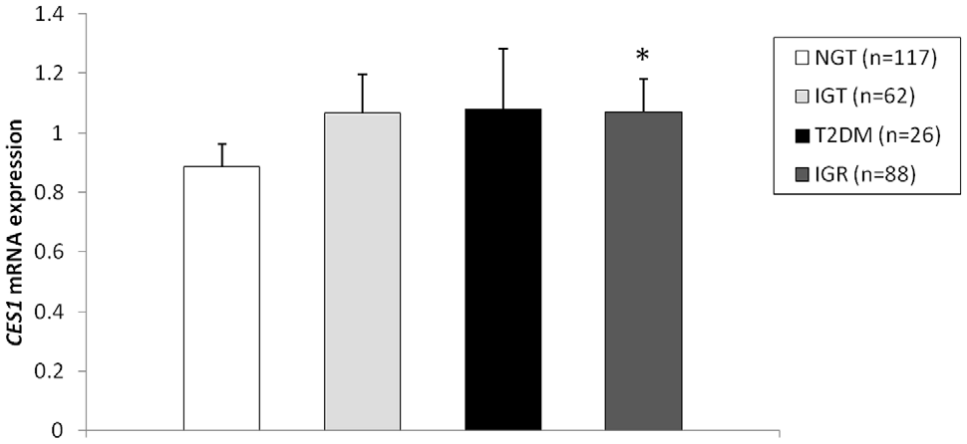
*CES1* mRNA expression level in adipose tissue in individuals with normal glucose tolerance (NGT), impaired glucose tolerance (IGT), type 2 diabetes mellitus (T2DM), and impaired glucose regulation (IGR = IGT+T2DM). *CES1*: carboxylesterase 1 gene. *P<0.05 compared to NGT after adjustment for age, gender, and body-mass index.

*CES1 and CES1P1 mRNA expression. CES1* mRNA expression level in adipose tissue was significantly affected by age, gender, and BMI (*P*<0.001 for all variables, Table 1). One year of aging was associated with a decrease in the *CES1* mRNA level of 4%, and the expression level in males was 35% lower than in females. Adiposity was associated with a 10% increase in *CES1* mRNA level per BMI unit (Table 1 and Figure 1A). Moreover, we found *CES1* mRNA level in adipose tissue to be positively associated with HOMA-IR (Table 2 and Figure 1B), where a doubling of this level was associated with a 15% increase in HOMA-IR (*P* = 0.003). *CES1* mRNA level was also positively associated with other measures of metabolic function (Table 2), including cholesterol (*P* = 0.04), and fasting plasma levels of insulin (*P* = 0.006), glucose (*P* = 0.002) and triglycerides (*P* = 0.003, see also Figure 1C). Conversely, *CES1* mRNA level was negatively associated with ISI_composite_ (*P* = 0.005). Finally, there was a positive correlation between the levels of mRNA of *CES1* and *CES1P1* (Figure 1D). Although *CES1* mRNA level was not significantly associated with 120-min OGTT glucose level (Table 2), we found a borderline significant association (*P* = 0.057) between the levels of OGTT glucose and *CES1* mRNA after adjustment for age, sex and BMI (Figure 2). Individuals with IGT and T2DM were combined into one group with impaired glucose regulation. After adjustment for age, sex and BMI the level of *CES1* mRNA was 27% higher in the combined group compared with the NGT group (Figure 2).

**Table 1:** Effect of selected variables on *CES1* mRNA expression level in adipose tissue.

Explanatory variable	Regression coefficient	*P*-value
Age (effect of 1 year)	−4%	<0.001
Gender (effect of the male gender)	−35%	<0.001
BMI (effect of 1 BMI unit)	+10%	<0.001

The statistical model is: ln(*CES1* mRNA level) = age+gender+BMI. The regression coefficient expresses the change in the response variable associated with an increase of one unit in an explanatory variable. *CES1*: carboxylesterase 1 gene; BMI: body-mass index.

**Table 2:** Association between *CES1* mRNA expression level in adipose tissue and measures of glucose and lipid metabolism.

Response variable	Regression coefficient	*P*-value
HOMA-IR	+15%	0.003
ISI_composite_	−10%	0.005
120-min glucose, OGTT	+3%	0.23
Fasting triglyceride level	+10%	0.003
Fasting insulin level	+12%	0.006
Fasting glucose level	+3%	0.002
Total cholesterol level	+3%	0.04

The statistical model is: ln(response variable) = age+sex+BMI+*CES1* mRNA level. The regression coefficient expresses the change in the response variable associated with a doubling of *CES1* mRNA level from its average. *CES1*: carboxylesterase 1 gene; HOMA-IR: homeostasis model assessment-insulin resistance; ISI_composite_: insulin sensitivity index (composite); OGTT: oral glucose tolerance test.

### Heritability of CES1 and CES1P1 mRNA Expression

The heritability coefficients for the mRNA expression of *CES1* and *CES1P1* amounted to ∼1 and 0.84, respectively. This suggested that genetic effects exhibited a quantitatively predominant influence on the variation of the mRNA expression of these two genes in adipose tissue (Table 3).

**Table 3:** Heritability of *CES1* and *CES1P1* mRNA expression levels in adipose tissue.

Variable	r_MZ_	r_DZ_	h^2^
*CES1*	0.82 (n = 23 pairs)	0.26 (*n* = 32 pairs)	∼1
*CES1P1*	0.78 (*n* = 14 pairs)	0.36 (*n* = 24 pairs)	0.84

The heritability was determined as: h^2^ = 2[r_MZ_−r_DZ_]. In this equation h^2^ is the heritability coefficient; r_MZ_ and r_DZ_ are intra-class correlations within monozygotic (MZ) and dizygotic (DZ) twins, respectively. Due to missing biopsies or technical issues we only determined *CES1* and *CES1P1* mRNA level in a subgroup of the twin subjects. *CES1*: carboxylesterase 1 gene; *CES1P1*: carboxylesterase 1 pseudogene 1.

*CES1 copy number variation.* Since the *CES1* mRNA expression level in adipose tissue was found to be highly heritable and significantly associated with numerous measures of metabolic function, we evaluated the effect of *CES1* duplication, a genetic variation that previously has been suggested to affect the transcript abundance of this gene [16]. Among the subjects in the study population, 6 carried the duplicated allele on both chromosomes, 60 were heterozygotes for this allele, and 138 did not carry the duplicated allele. Based on this observation, the frequency of the duplicated *CES1* allele was estimated at 0.18. The genotype proportions conformed to those expected under the condition of Hardy-Weinberg equilibrium (*P* = 0.86). Due to the small number of subjects having 4 gene copies of *CES1*, we amalgamated the groups with 3 and 4 copies of *CES1* (Table 4). Using this approach we found decreases of 5% and 14% in the fasting level of glucose (*P* = 0.06) and insulin (*P* = 0.04), respectively, in the group of subjects with 3 or 4 *CES1* gene copies, while there was a 9% increase in ISI_composite_ (*P* = 0.05) in this group compared with individuals harboring 2 copies of the gene (Table 4). Moreover, we found a 19% decrease in of HOMA-IR (*P* = 0.02) and an 11% decrease in OGTT (*P* = 0.03) in the group of subjects with 3 or 4 *CES1* gene copies. There was no significant association between the gene copy number and *CES1* mRNA expression (*P* = 0.63). Furthermore, *CES1* copy number was not associated with measures of adiposity or plasma lipid parameters, including plasma triglyceride and total cholesterol levels. Statistical analysis without amalgamation of the groups with 3 and 4 *CES1* copies produced results that were comparable to those obtained with amalgamation. However, a larger effect of *CES1* copy number was observed on HOMA-IR (*P* = 0.002) and level of fasting insulin (*P* = 0.002) without the amalgamation.

**Table 4:** Association of *CES1* gene copy number with measures of adiposity and metabolic regulation.

*CES1* copy number	2^1^	3 or 4^1^	*P*-value
Age (years)	73 (5)	73 (5)	
*n* (male/female)	168 (81/87)	74 (28/46)	
BMI	25.9 (3.5)	26.3 (4.1)	0.44
WHR	0.90 (0.10)	0.89 (0.10)	0.99
Fasting TG (mM)	1.2 (0.6)	1.3 (0.6)	0.66
Fasting insulin (pM)	50 (34)	43 (20)	0.04
Fasting glucose (mM)	5.9 (1.1)	5.6 (0.6)	0.06
HOMA-IR	1.9 (1.5)	1.6 (0.8)	0.02
ISI_composite_	15.5 (7.7)	17.1 (8.0)	0.05
120-min glucose, OGTT (mM)	8.7 (4.3)	7.4 (2.2)	0.03
Total cholesterol (mM)	5.5 (1.0)	5.7 (0.8)	0.07
LDL (mM)	3.4 (2.5)	3.3 (0.8)	0.96
VLDL (mM)	0.60 (0.50)	0.60 (0.26)	0.97
*CES1* mRNA level	0.98 (0.92)	0.92 (0.83)	0.63
*CES1P1* mRNA level	1.9 (2.2)	1.0 (1.0)	0.003

The statistical model is: ln(response variable) = age+sex+BMI+*CES1* copy number.

1 Group average with SD in brackets. BMI: body-mass index; *CES1*: carboxylesterase 1 gene; *CES1P1*: carboxylesterase 1 pseudogene 1; HOMA-IR: homeostasis assessment model-insulin resistance; ISI_composite_: insulin sensitivity index (composite); LDL: low density lipoprotein; OGTT: oral glucose tolerance test; TG: triglycerides; VLDL: very low density lipoprotein; WHI: waist-hip ratio.

## Discussion

In the present study the mRNA expression level of *CES1* in subcutaneous adipose tissue samples derived from monozygotic and dizygotic twins was examined. We report several important findings about the regulation and possible role of CES1 in the lipid and glucose homeostasis. First, we found significant relationships between *CES1* mRNA level and age, gender, measures of adiposity, metabolic regulation and *CES1P1* mRNA level. Second, a high heritability for the level of mRNA expression of *CES1* was detected. Third, *CES1* gene copy number was found to be associated with measures of glucose metabolism. Collectively, these findings suggest that CES1 is involved in the pathogenesis of T2DM.

Our study is the first to report that *CES1* mRNA level in subcutaneous adipose tissue is negatively correlated with age in a population of elderly subjects, and that *CES1* is transcribed at significantly higher levels in women than men. Although a previous small-sized study also found *CES1* mRNA expression level in visceral adipose tissue to be inversely associated with age, the association for subcutaneous adipose tissue in this study did not reach statistical significance. However, since the age span in the present study was only 21 years with the youngest subject being 62 years old, it cannot be excluded that age affects *CES1* mRNA level in adipose tissue differently in younger individuals. The strong correlations of *CES1* mRNA level with BMI, total cholesterol, and fasting plasma glucose and insulin levels observed in the present study are remarkable and may clarify disagreeing findings observed in previous studies [13], [14]. For example, one study reported lack of association of *CES1* mRNA level with BMI [13], while another study found a strong positive correlation between these two variables [14]. Based upon a large sample our findings support the latter of these two studies. The finding that individuals with dysregulated glucose metabolism, including those with T2DM, displayed higher levels of *CES1* mRNA underscores the association of *CES1* with glucose metabolism. Our finding of a positive correlation between the expression level of mRNA of *CES1P1* and *CES1* is interesting and since pseudogenes can affect the levels of mRNA of their protein-coding counterparts [17], [27], [28], it suggests a novel mechanism for regulation of the *CES1* mRNA expression in adipose tissue. Previous observations have suggested that the transcripts of a pseudogene and its functional counterpart can compete for the same transacting factors involved in mRNA degradation [27]. Therefore, high levels of transcripts generated from a pseudogene may efficiently protect the transcripts of a functional homolog from degradation, thus increasing its translation. Whether such mechanism is involved in the regulation of CES1 activity is not known. Of importance, a pseudogene has previously been associated with insulin resistance and T2D by another posttransciptional regulation mechanism that involved destabilization of the transcripts generated from a functional homolog [29].

There are several plausible explanations for the observed associations between the level of *CES1* mRNA in subcutaneous adipose tissue and measures of glucose metabolism. Notably, increased CES1-mediated lipolysis in adipose tissue could cause disturbances of the glucose metabolism by elevation of the plasma levels of non-esterified fatty acids that can induce muscle and hepatic insulin resistance [30], [31] Also, cholesterol imbalance in adipocytes seems capable of inducing insulin resistance [2] and since CES1 is believed to have an important role in regulating the ratio of esterified and non-esterified cholesterol [32], such regulating role of CES1 could be reflected in the appearance of an association between levels of *CES1* mRNA and cholesterol. Alternatively, imbalance in the metabolism of cholesterol or other lipids due to intake of a diet rich in animal fat may lead to an altered blood lipid profile [33], [34] and increments in body fat deposits [35] that might affect the expression of *CES1* mRNA.

In the present study, the heritability of *CES1* mRNA expression in subcutaneous adipose tissue was high, exceeding the average heritability of 0.234 for the mRNA expression across genes in subcutaneous adipose tissue previously obtained by analysis of a family cohort [36]. Heritability coefficients have several limitations. For example, they can be inflated by shared pre- as well as post-natal environments [37]. However, even with this limitation the present study suggests the existence of a strong heritable component in the expression of *CES1* mRNA in adipose tissue. A strong heritable component in the transcription is characteristic of genes whose transcriptional levels are determined by local regulatory variation [38], [39]. Hence, the individual differences in the expression level of *CES1* mRNA observed in the present study may to large extent reflect variation within this gene or in genes in its vicinity.

A genetic variation potentially conferring individual differences in the expression of *CES1* is duplication of the gene [15], [16]. Previously, the frequency of the duplicated allele of *CES1* was determined at 0.20 among voluntary blood donors in Denmark (Rasmussen et al., unpublished results). Although slightly lower, the observed frequency of this allele in the present study, based upon related as well as unrelated subjects, was in accordance with this value. Apparently, the transcription in adipose tissue by genotypes with 3 or 4 copies of *CES1* was comparable with that of genotypes composed of 2 copies of the gene. Probably, this reflects that the “daughter” copy, often designated *CES1A2*, is transcribed at a low level and therefore does not contribute substantially to the total amounts of *CES1* transcripts. This is in agreement with findings obtained by analysis of human livers [15], while the expression of *CES1A2* has been found to be high in several cell lines [16]. In addition to gene duplication, variation in the promoter region of a gene can affect the amounts of transcripts produced. There are several promoter variants in *CES1* with a presumed effect on the transcription of this gene [40], [41]. However, these effects remain to be confirmed.

The copy number of the gene encoding CES1, including “mother” as well as “daughter” copies, was associated with several measures of glucose metabolism. More specifically, presence of 2 copies of *CES1* appeared to predispose to a negative phenotype and influence the susceptibility to T2DM suggesting that the present study identified a novel genetic factor affecting the risk of developing this disorder. In this regard, the lack of association between *CES1* gene copy number variation and mRNA level in adipose tissue is surprising, when this genetic variation is proposed to have effects on glucose and lipid metabolisms. One explanation for this apparent discrepancy may be that the duplicated version of *CES1* is in linkage disequilibrium with a gene variant that affects glucose metabolism indirectly or directly without increasing the expression of *CES1* mRNA. An alternative possibility is that CES1 activity in adipose tissue is predominantly regulated by posttranscriptional mechanisms including pseudogene-mediated transcript protection, a mechanism that appears to play a role in the regulation of several other genes [17], [27], [28]. This is an interesting possibility since the haplotype harboring the duplicated allele lacks *CES1P1*, while this pseudogene is present on the haplotype with the unduplicated allele [15]. Therefore, the latter of these two haplotypes has a potential for generation of *CES1P1* transcripts.

Pseudogenes may act through different mechanisms [17], [27], [28]. Notably, antisense transcripts generated from these genetic elements have been implicated in the translational silencing of their functional homologs [17], [28]. It is possible that pseudogen-mediated posttranscriptional regulation is involved in determining CES1 activity and that this explains some of the findings from the present study. Regulation of the activity of CES1 may, however, be even more complex as it also seems to include regulatory control on the protein level by endogenous small molecules, such as cholesterol-like molecules [32]. Therefore, CES1 activity may not be correlated with level of *CES1* mRNA and copy number status of *CES1*.

Various aspects of the role of CES1 in lipid metabolism remain to be clarified including the importance of CES1-mediated lipolysis in adipose tissue relative to that in the liver. A recent study raised doubts about the importance of CES1 in the hydrolysis of triglycerides in adipose tissue and suggested that other lipases were more important in this respect [12], while another study reported a major role of CES1 in the trafficking of lipids and lipid metabolism in hepatocytes [42]. Whether CES1 is more important to the triglyceride metabolism in the liver than adipose tissue of humans is not yet known. However, using mice with global knock-out of Ces1d1, the murine ortholog of the human CES1, a recent study attributed most of the reduction in plasma triglycerides resulting from this gene knock-out to the absence of hepatic activity of Ces1d1 [9]. If CES1 has a role in the metabolic regulation in the liver that is comparable to its murine ortholog, this would explain our finding that *CES1* gene copy number was associated with insulin resistance in the fasting state (HOMA-IR), which is thought to be primarily hepatically determined [43].

The positive association between *CES1* gene copy number and ISI_composite_ suggests that variations in this gene impacts on the peripheral insulin sensitivity [23], possibly affecting glucose metabolism or insulin sensitivity in skeletal muscle. Although it is known that insulin sensitivity is under strong genetic control [44], [45], very few genetic variations affecting insulin sensitivity in the liver or skeletal muscle have been identified so far. Our results, suggest that copy number variation of *CES1* could be involved in mediating such effects. Finally, besides the association with ISI_composite_, *CES1* copy number was associated with glucose tolerance, a positive association that might reflect an effect on beta cell function and insulin secretion.

In conclusion, the present study links the expression level of *CES1* mRNA with risk factors for T2DM and suggests that copy number variation of *CES1* influences measures of glucose metabolism, perhaps contributing to the genetic susceptibility to T2DM. This knowledge may be valuable for future research aimed at understanding the genetic architecture predisposing for T2DM.
